# Supplementary material for: Statin-dye conjugates for selective targeting of KRAS mutant cancer cells
Source: PLoS One. 2026 Jan 9;21(1):e0340189. doi: 10.1371/journal.pone.0340189 (PMC12788682; doi:10.1371/journal.pone.0340189)
Supplement: S1 Fig — Conjugation was verified by the appearance of ester-specific methylene (1H: δ 5.20-5.06 ppm) and carbonyl (13C: δ 176.4-168.6 ppm) signals. (PDF) [file pone.0340189.s001.pdf]

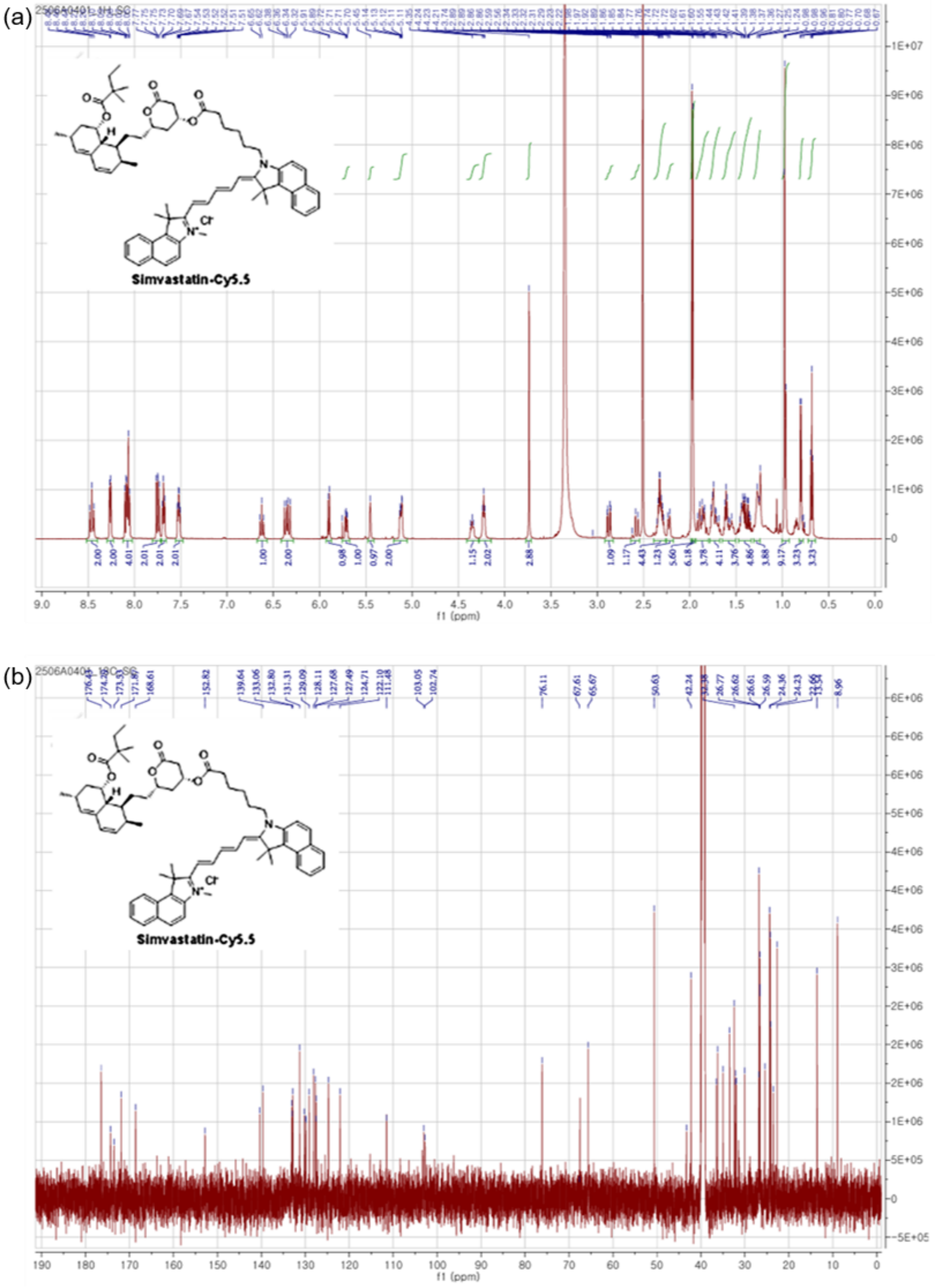

Figure S1. Chemical structure of simvastatin-Cy5.5 was confirmed *via* (a) ^{1}H-NMR and (b) ^{13}C-NMR, respectively. Conjugation was verified by the appearance of ester-specific methylene (^{1}H: δ 5.20-5.06 ppm) and carbonyl (^{13}C: δ 176.4-168.6 ppm) signals.
