# Supplementary figures and images for: Statin-dye conjugates for selective targeting of KRAS mutant cancer cells
Source: PLoS One. 2026 Jan 9;21(1):e0340189. doi: 10.1371/journal.pone.0340189 (PMC12788682; doi:10.1371/journal.pone.0340189)

(a)

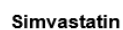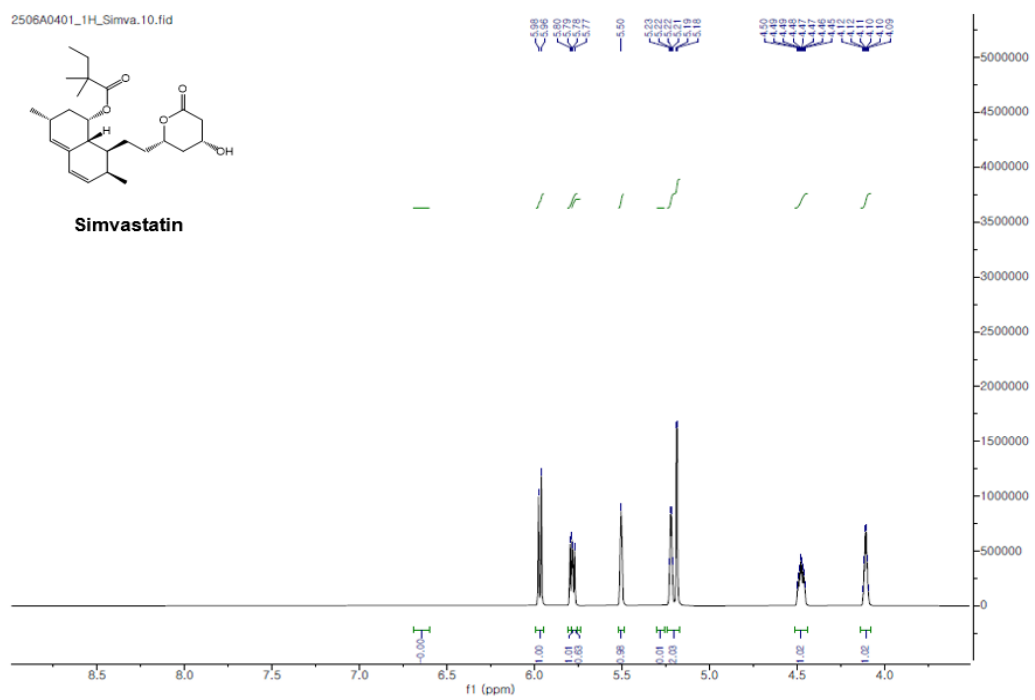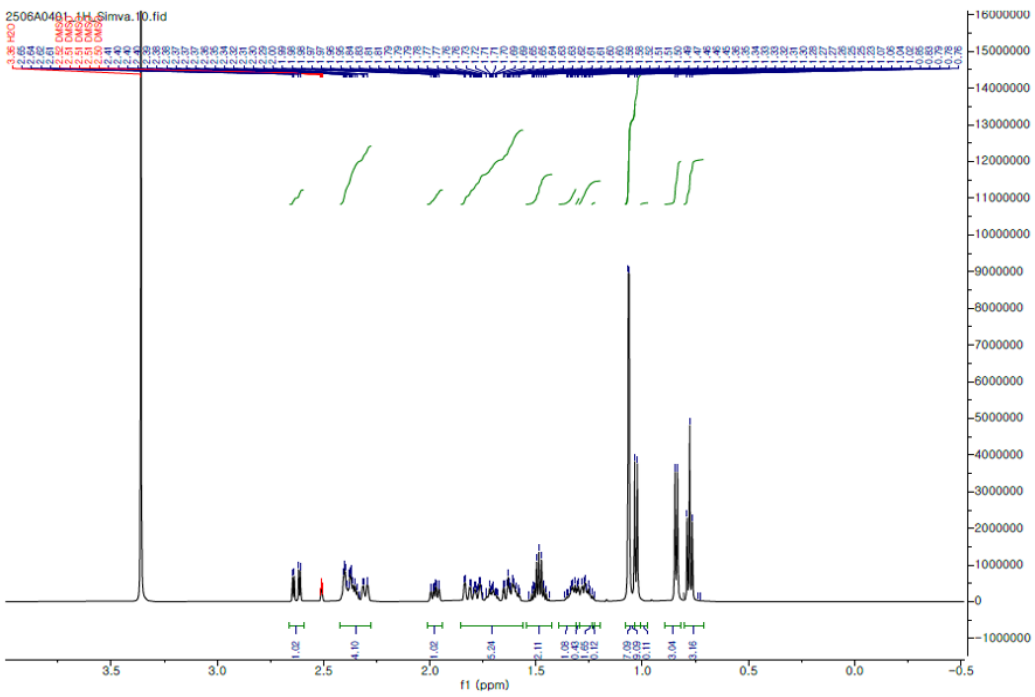

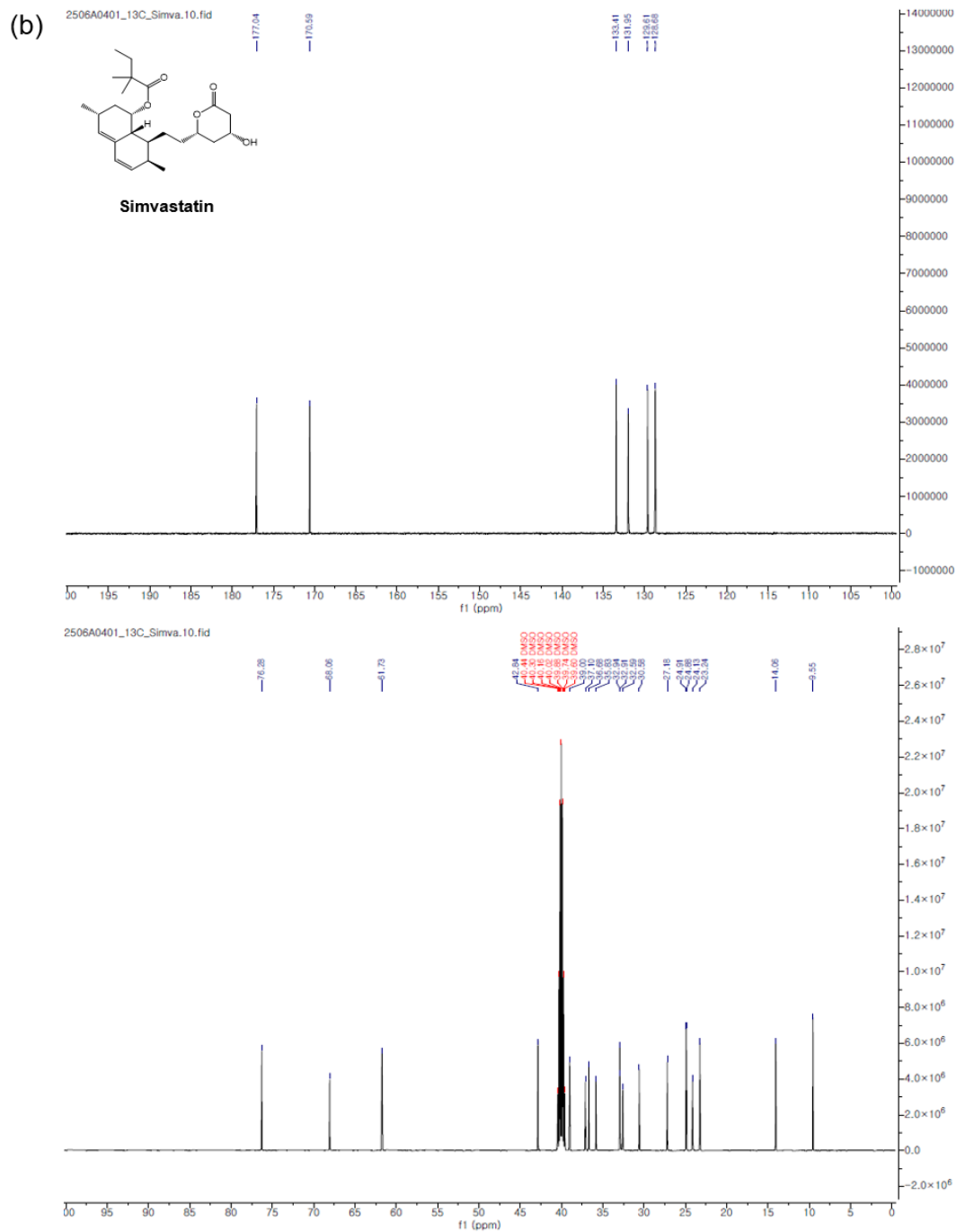

(c) 2506A0401\_1H\_Prava.10.fid

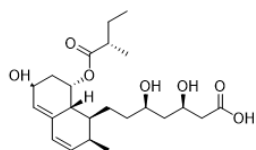

Pravastatin

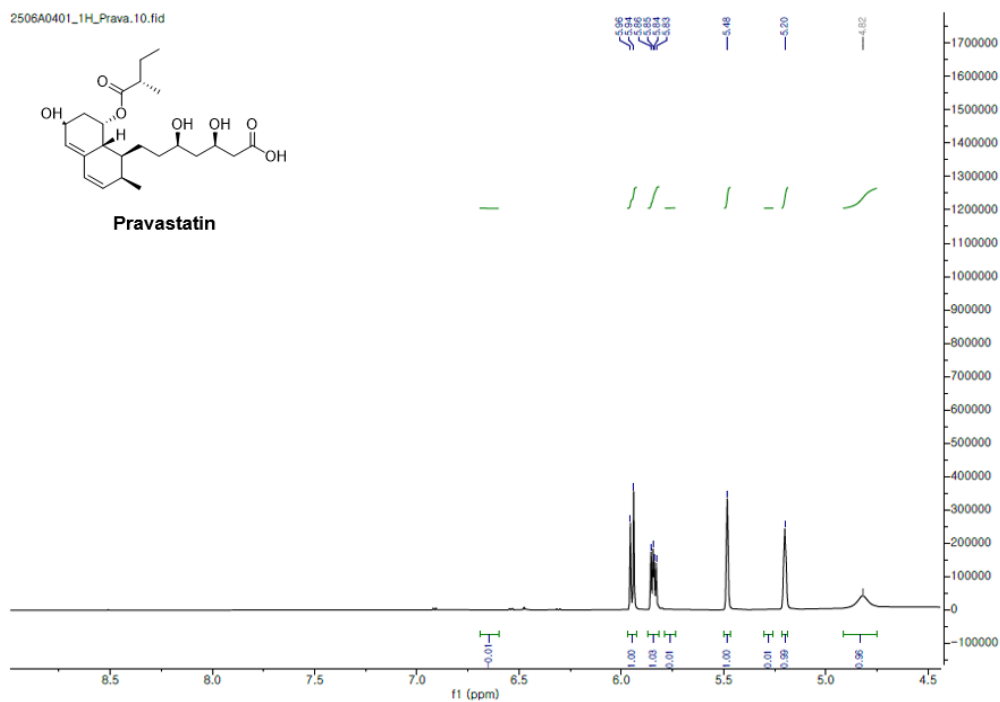

2506A0401\_1H\_Prava.10.fid

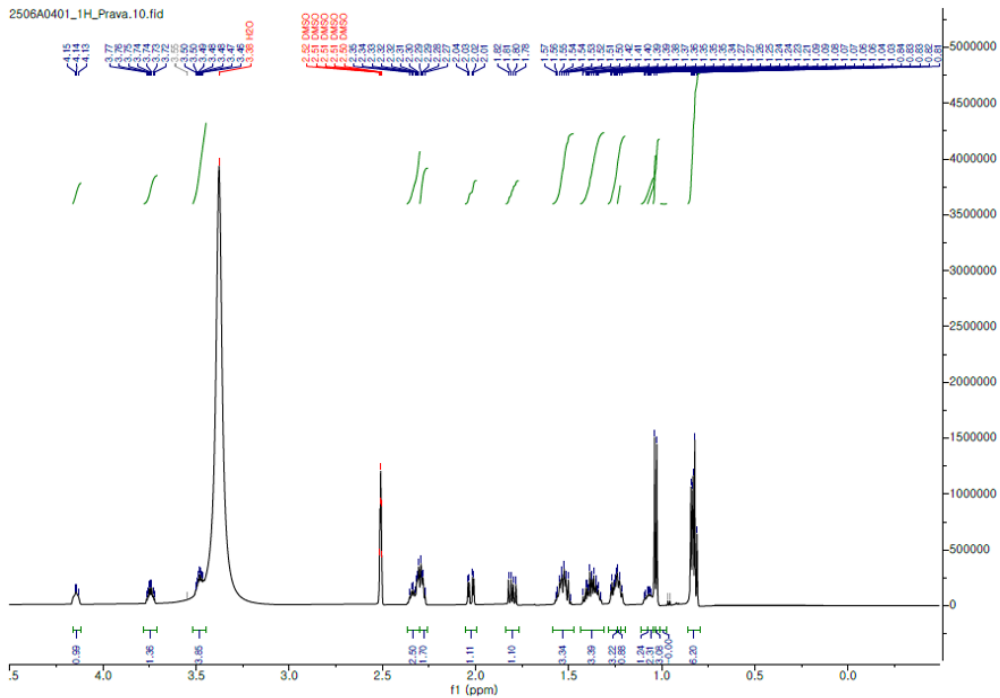

(d)

2506A0401\_13C\_Prava.10.fid

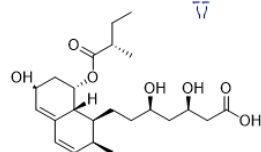

Pravastatin

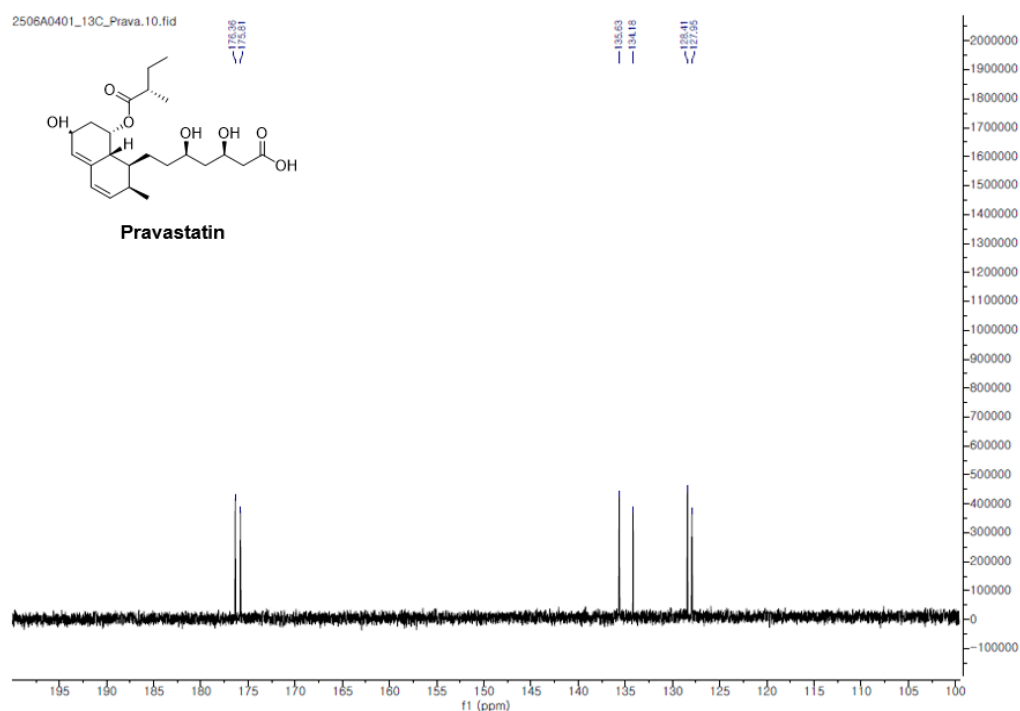

2506A0401\_13C\_Prava.10.fid

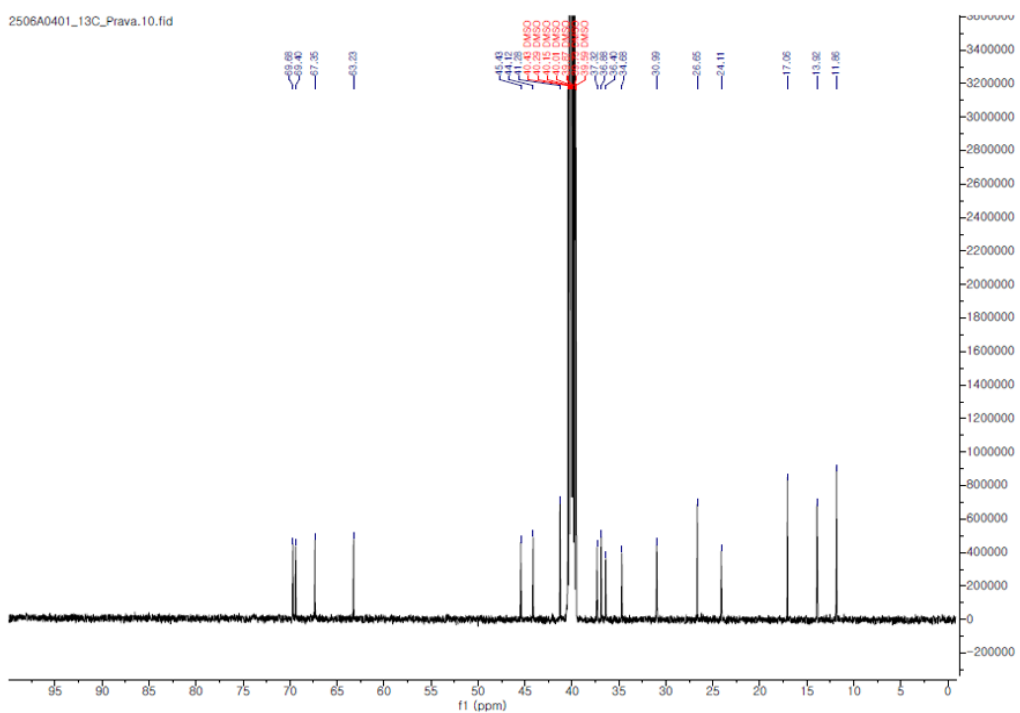

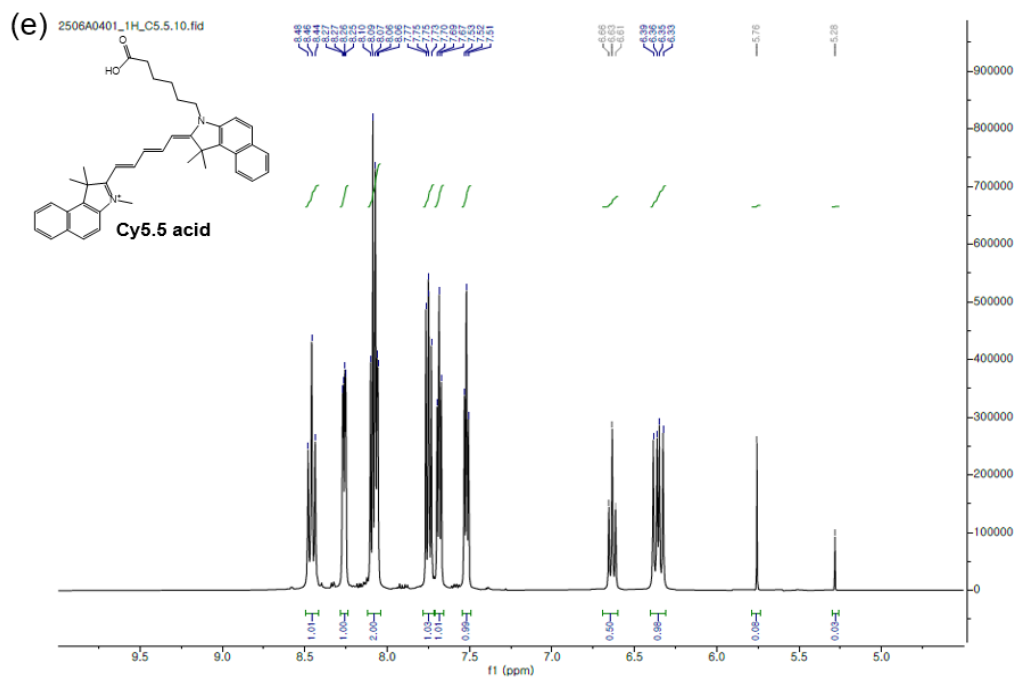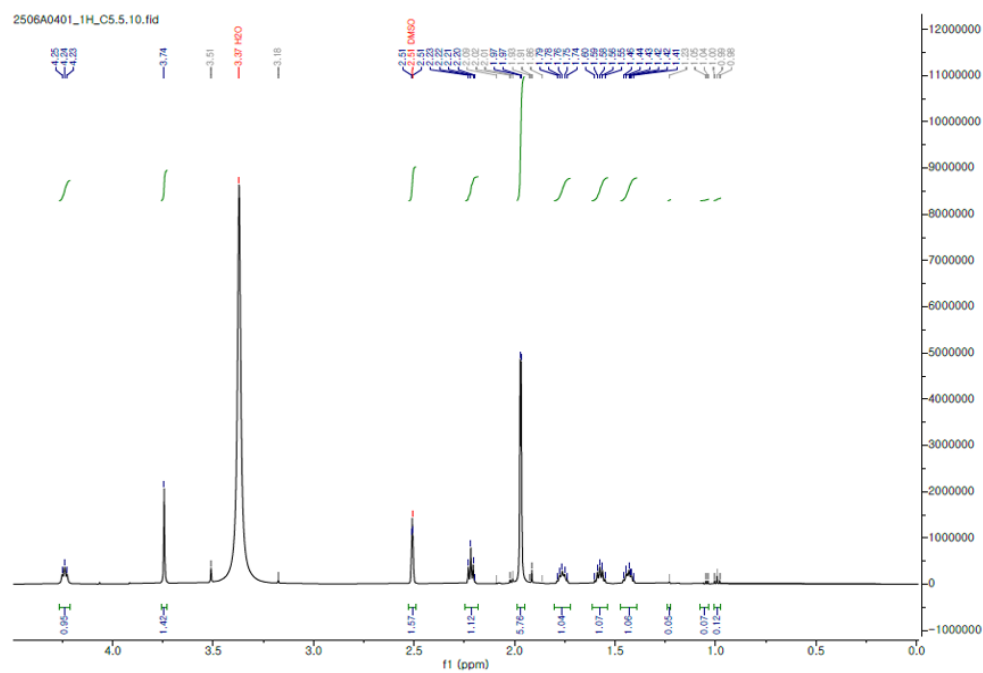

Supplement: S3 Fig — (PDF) [file pone.0340189.s003.pdf]
