# Supplementary material for: Statin-dye conjugates for selective targeting of KRAS mutant cancer cells
Source: PLoS One. 2026 Jan 9;21(1):e0340189. doi: 10.1371/journal.pone.0340189 (PMC12788682; doi:10.1371/journal.pone.0340189)
Supplement: S4 Fig — Confocal microscopy images show the cellular uptake of simvastatin-Cy5.5 (a) and Cy5.5 alone (b) in KRASMUT Panc1 cells at two different concentrations: 50 nM and 17 nM. Nuclei were stained with DAPI (blue), and Cy5.5 fluorescence is shown in red. Simvastatin-Cy5.5 exhibited concentration-dependent uptake, with higher fluorescence intensity observed at 50 nM compared to 17 nM. In contrast, Cy5.5 alone showed minimal uptake at both concentrations, suggesting that the statin moiety facilitates selective internalization in KRASMUT cells. The scale bars indicate 500 μm. (PDF) [file pone.0340189.s004.pdf]

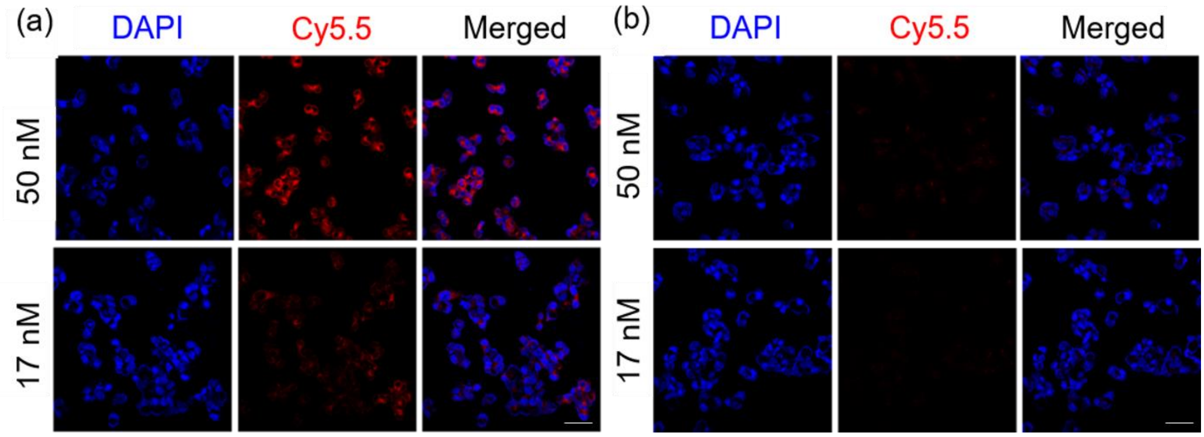

Figure S4. Concentration-dependent uptake of simavastatin-Cy5.5 in *KRAS*-mutant (*KRAS*^{MUT}) Panc1 cells. Confocal microscopy images showing the cellular uptake of simvastatin-Cy5.5 (a) and Cy5.5 alone (b) in *KRAS*^{MUT} Panc1 cells at two different concentrations: 50 nM and 17 nM. Nuclei was stained with DAPI (blue), and Cy5.5 fluorescence is shown in red. Simvastatin-Cy5.5 exhibited concentration-dependent uptake, with higher fluorescence intensity observed at 50 nM compared to 17 nM. In contrast, Cy5.5 alone showed minimal uptake at both concentrations, suggesting that the statin moiety facilitates selective internalization in *KRAS*^{MUT} cells. The scale bars indicate 500 µm.
