# Supplementary material for: Statin-dye conjugates for selective targeting of KRAS mutant cancer cells
Source: PLoS One. 2026 Jan 9;21(1):e0340189. doi: 10.1371/journal.pone.0340189 (PMC12788682; doi:10.1371/journal.pone.0340189)
Supplement: S5 Fig — (a) Cellular uptake of simvastatin-Cy5.5, pravastatin-Cy5.5, and Cy5.5 in Panc1 cells measured by flow cytometry. Bars indicate Mean ± S.E. (n ≥ 3). Statistically significant differences are represented as **** for p < 0.0001. Cellular uptake of simvastatin-Cy5.5 (red) in isogenic (b) HCT116 and (c) DLD1 cells. The scale bars indicate 100 μm. The cell nuclei were stained with DAPI (blue). (PDF) [file pone.0340189.s005.pdf]

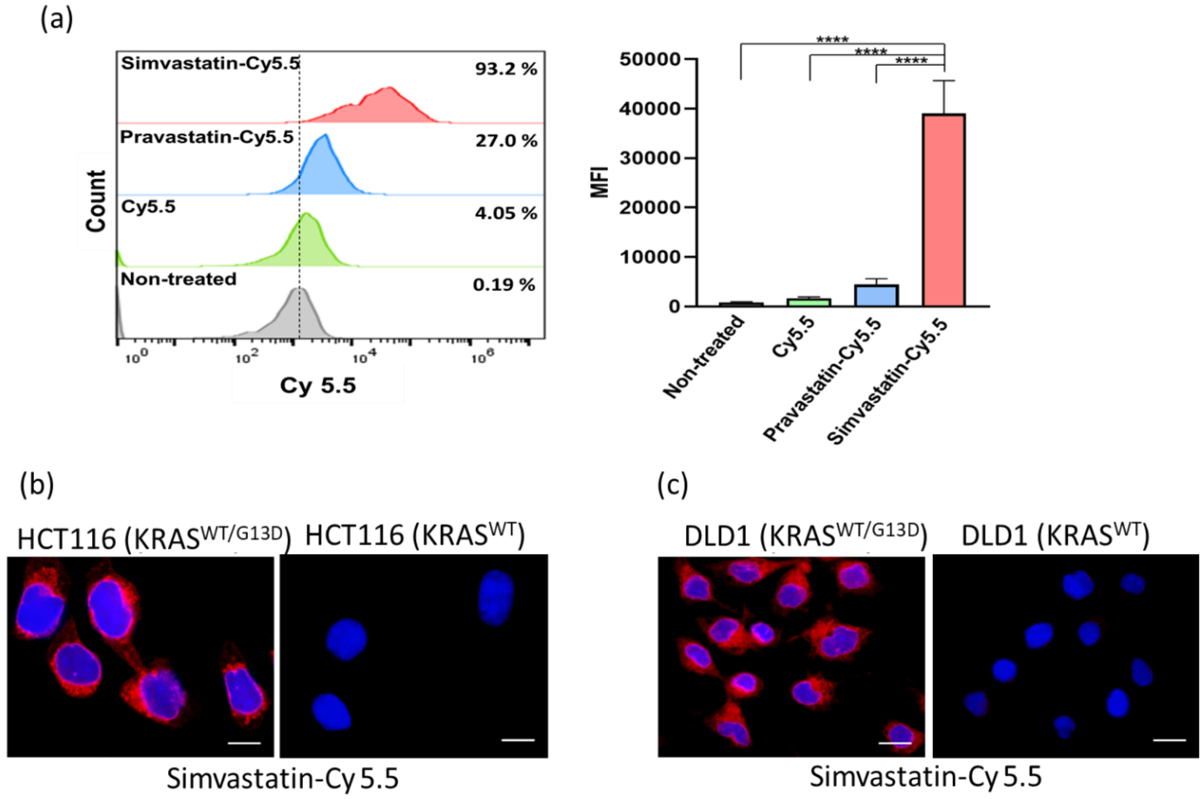

Figure S5. Enhanced cellular uptake of simvastatin-Cy5.5 in *KRAS*^{MUT} cancer cells. (a) Cellular uptake of simvastatin-Cy5.5, pravastatin-Cy5.5, and Cy5.5 in Panc1 cells measured by flow cytometry. Bars indicate Mean ± S.E. ($n \geq 3$). Statistically significant differences are represented as **** for $p < 0.0001$. Cellular uptake of simvastatin-Cy5.5 (red) in isogenic (b) HCT116 and (c) DLD1 cells. The scale bars indicate 100 μm. The cell nuclei were stained with DAPI (blue).
