# Supplementary material for: Statin-dye conjugates for selective targeting of KRAS mutant cancer cells
Source: PLoS One. 2026 Jan 9;21(1):e0340189. doi: 10.1371/journal.pone.0340189 (PMC12788682; doi:10.1371/journal.pone.0340189)
Supplement: S6 Fig — (a) Schematic diagram illustrating the synthesis of the PEG-Cy5.5 conjugate. SPA: Succinimidyl propionate; DMSO: Dimethylsulfoxyde. (b) High-performance liquid chromatography (HPLC) chromatograms of Cy5.5 amine and PEG-Cy5.5. (PDF) [file pone.0340189.s006.pdf]

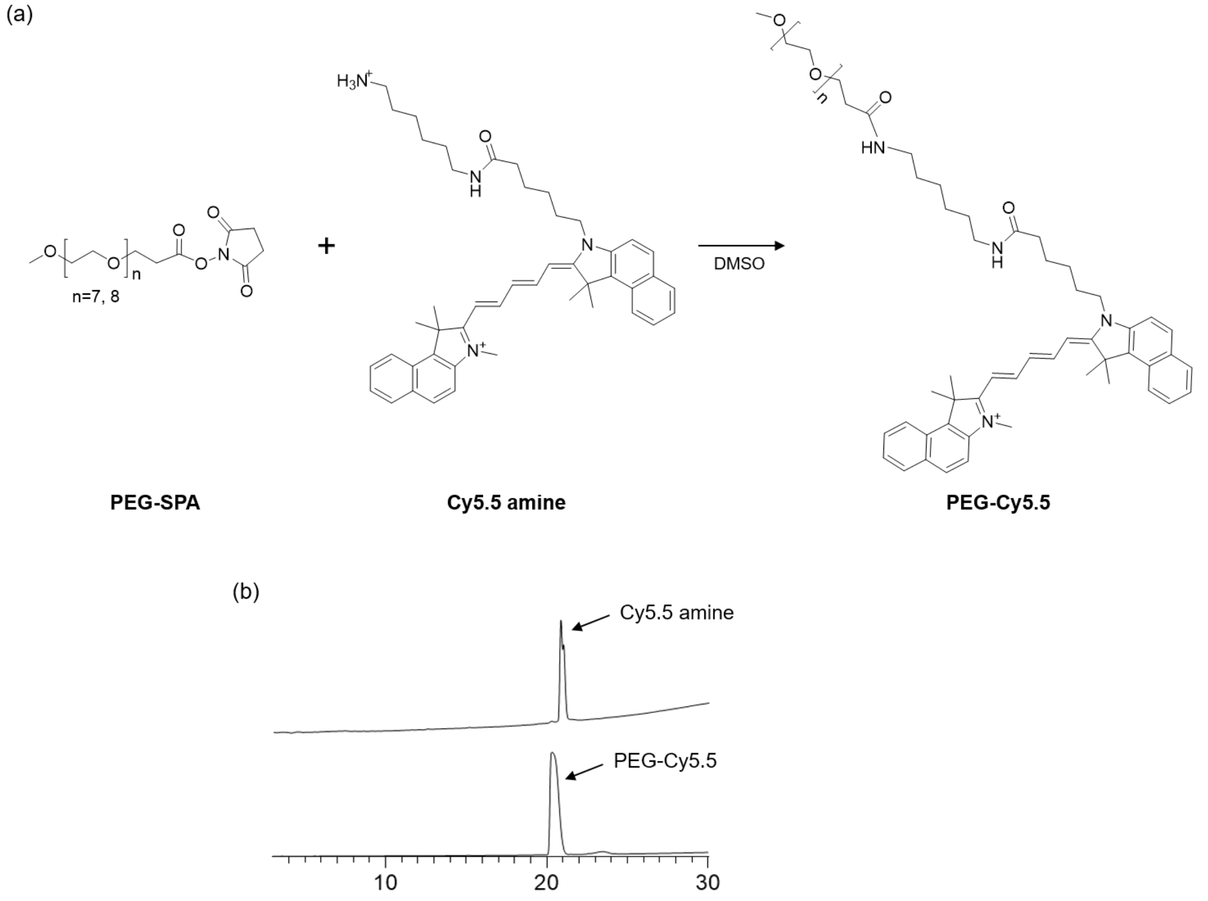

Figure S6. Synthesis and characterization of PEG-Cy5.5. (a) Schematic diagram illustrating the synthesis of the PEG-Cy5.5 conjugate. SPA: Succinimidyl propionate; DMSO: Dimethyl sulfoxide. (b) High-performance liquid chromatography (HPLC) chromatograms of Cy5.5 amine and PEG-Cy5.5.
