# Supplementary material for: Statin-dye conjugates for selective targeting of KRAS mutant cancer cells
Source: PLoS One. 2026 Jan 9;21(1):e0340189. doi: 10.1371/journal.pone.0340189 (PMC12788682; doi:10.1371/journal.pone.0340189)
Supplement: S7 Fig — (a) Confocal images showing the cellular uptake of Cy5.5 acid, Cy5.5 amine, PEG-Cy5.5 and simvastatin-Cy5.5 in Panc1 cells. Cells were incubated with 50 nM of each molecule for 1 hour. Red fluorescence represents internalized Cy5.5 conjugates, and blue fluorescence (DAPI) indicates cell nuclei. The scale bars indicate 20 μm. (b) Corresponding quantification of mean fluorescence intensity (MFI) per cell. Data are presented as mean ± S.E. (n = 5 randomly selected images per group). Statistical significance was analyzed by one-way ANOVA; *p < 0.05, ****p < 0.0001. (PDF) [file pone.0340189.s007.pdf]

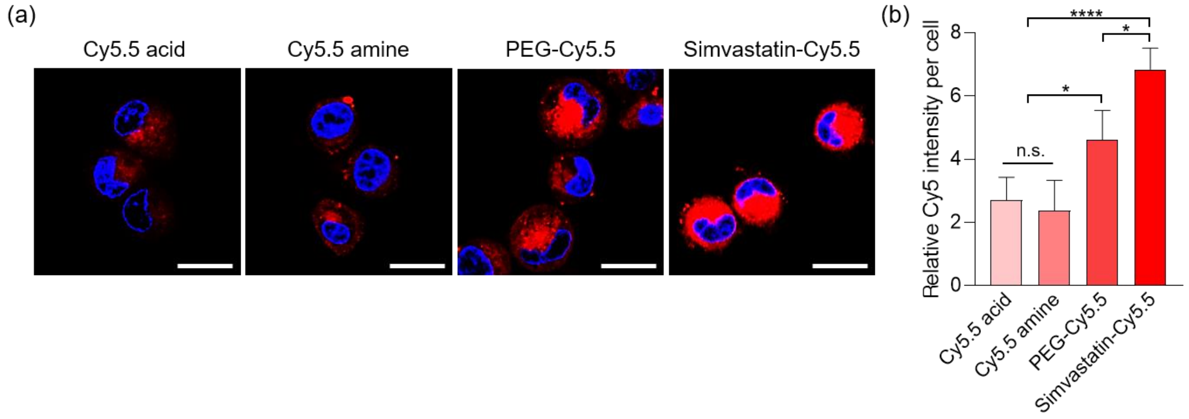

Figure S7. Cellular uptake of Cy5.5 conjugates in KRAS^{MUT} cancer cells. (a) Confocal images showing the cellular uptake of Cy5.5 acid, Cy5.5 amine, PEG-Cy5.5 and simvastatin-Cy5.5 in Panc1 cells. Cells were incubated with 50 nM of each molecule for 1 hour. Red fluorescence represents internalized Cy5.5 conjugates, and blue fluorescence (DAPI) indicates cell nuclei. The scale bars indicate 20 μm. (b) Corresponding quantification of mean fluorescence intensity (MFI) per cell. Data are presented as mean ± S.E. (n=5 randomly selected images per group). Statistical significance was analyzed by one-way ANOVA; *$p < 0.05$, ****$p < 0.0001$.
