# Supplementary material for: Statin-dye conjugates for selective targeting of KRAS mutant cancer cells
Source: PLoS One. 2026 Jan 9;21(1):e0340189. doi: 10.1371/journal.pone.0340189 (PMC12788682; doi:10.1371/journal.pone.0340189)
Supplement: S8 Fig — EIPA (50 μM) was pre-treated into cells for 1.5 h, followed by a 1 h incubation with fluorescein isothiocyanate-labeled BSA (FITC-BSA; 2 mg/mL), a widely used marker for macropinocytosis. Representative fluorescence images show FITC-BSA uptake (green) and nuclear staining with DAPI (blue). EIPA treatment significantly reduced FITC-BSA uptake in KRASMUT Panc1 cells, confirming its efficacy in inhibiting macropinocytosis. Additionally, Panc1 cells exhibited higher macropinocytosis activity compared to KRAS wild-type (KRASWT) BxPC3 cells. The scale bar indicates 100 μm. (PDF) [file pone.0340189.s008.pdf]

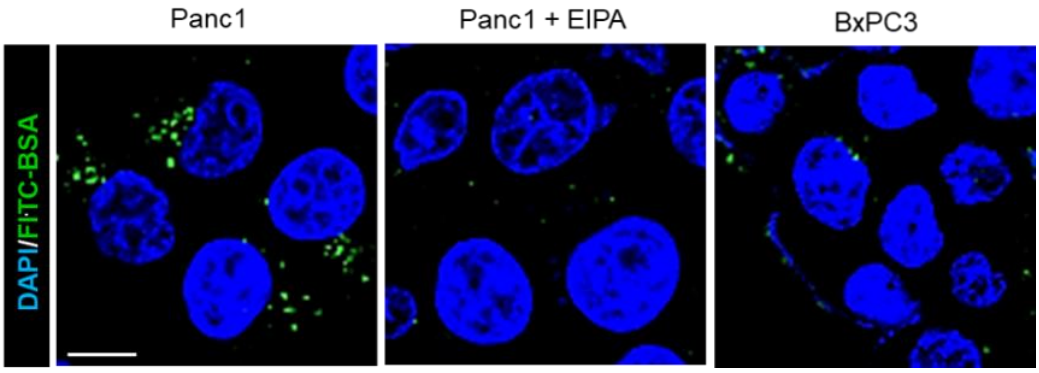

Figure S8. Validation of macropinocytosis inhibition by (5-(N-ethyl-N-isopropyl)amiloride (EIPA; macropinocytosis inhibitor) in *KRAS*^{MUT} Panc1 cells. EIPA (50 μM) was pre-treated into cells for 1.5 h, followed by a 1-h incubation with fluorescein isothiocyanate-labeled BSA (FITC-BSA; 2 mg/mL), a widely used marker for macropinocytosis. Representative fluorescence images show FITC-BSA uptake (green) and nuclear staining with DAPI (blue). EIPA treatment significantly reduced FITC-BSA uptake in *KRAS*^{MUT} Panc1 cells, confirming its efficacy in inhibiting macropinocytosis. Additionally, Panc1 cells exhibited higher macropinocytosis activity compared to *KRAS* wild-type (*KRAS*^{WT}) BxPC3 cells. The scale bar indicates 100 μm.
