# Supplementary material for: Statin-dye conjugates for selective targeting of KRAS mutant cancer cells
Source: PLoS One. 2026 Jan 9;21(1):e0340189. doi: 10.1371/journal.pone.0340189 (PMC12788682; doi:10.1371/journal.pone.0340189)
Supplement: S9 Fig — BKM120 (pan-PI3K inhibitor; 10 μM) was pre-treated into cells for 1 h and EIPA (macropinocytosis inhibitor; 50 μM) was pre-treated into cells for 1.5 h. The cell nuclei were stained with DAPI (blue). (PDF) [file pone.0340189.s009.pdf]

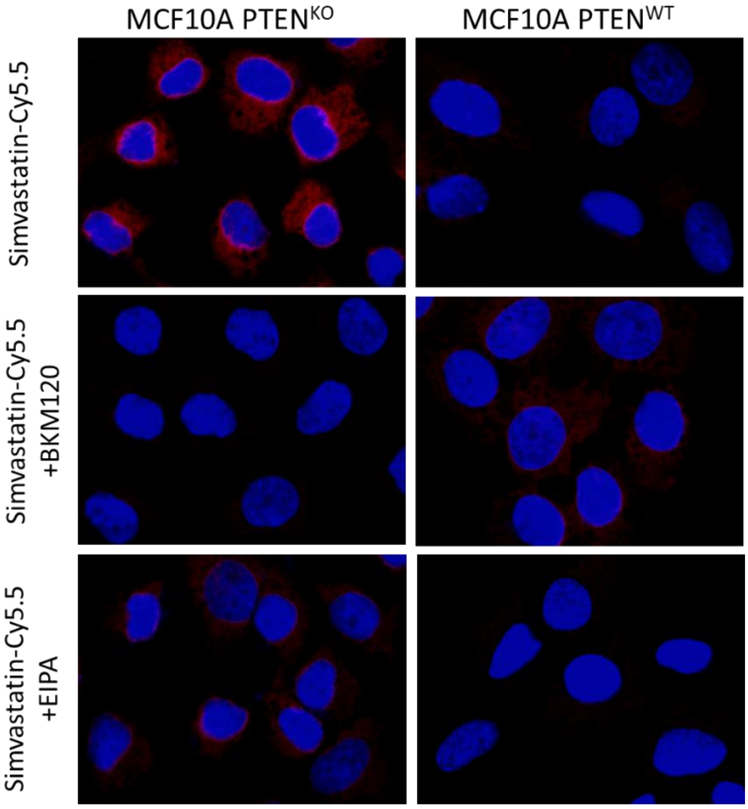

Figure S9. Cellular uptake of statin-Cy5.5 conjugates (red) by MCF10A cells with *PTEN* knockout (*PTEN*^{KO}) and wild-type (*PTEN*^{WT}). BKM120 (pan-PI3K inhibitor; 10 μM) was pre-treated into cells for 1 h and EIPA (macropinocytosis inhibitor; 50 μM) was pre-treated into cells for 1.5 h. The cell nuclei were stained with DAPI (blue).
