# Supplementary material for: Statin-dye conjugates for selective targeting of KRAS mutant cancer cells
Source: PLoS One. 2026 Jan 9;21(1):e0340189. doi: 10.1371/journal.pone.0340189 (PMC12788682; doi:10.1371/journal.pone.0340189)
Supplement: S10 Fig — Cell viability of CT26 and Panc1 cells after 24 h treatment with simvastatin-Cy5.5 (left, red) or unconjugated simvastatin (right, green) at concentrations of 0.1, 1, and 10 µM. While simvastatin-Cy5.5 induced dose-dependent cytotoxicity in KRASMUT cells, simvastatin alone showed minimal toxicity across the same dose range. Bars indicate mean ± S.E. (n ≥ 3). Statistical comparisons were made against the control group using Student’s t-test; *p < 0.05, **p < 0.01, ***p < 0.001. (PDF) [file pone.0340189.s010.pdf]

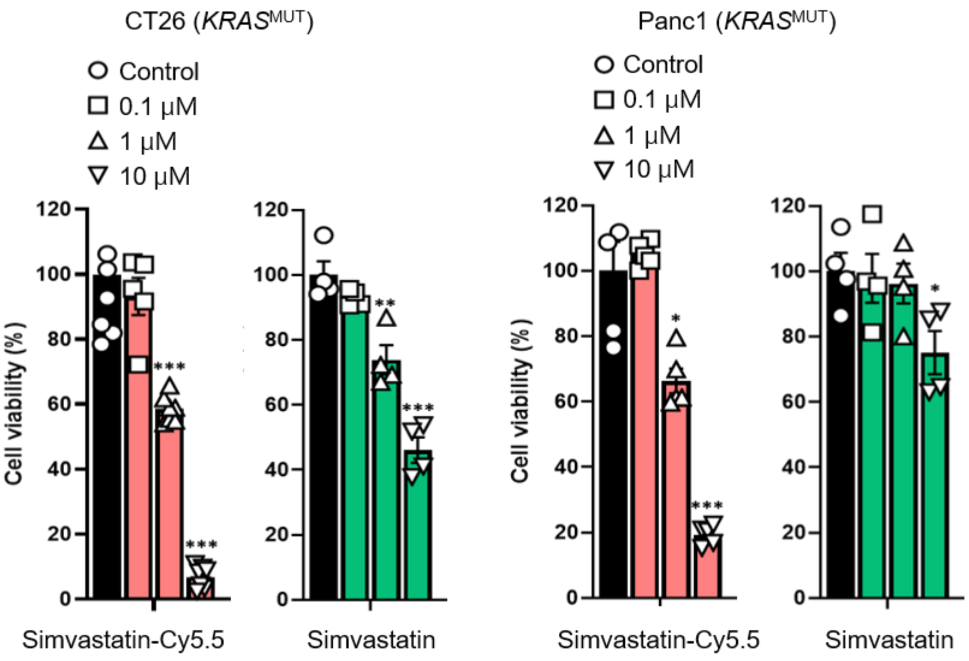

Figure S10. Selective cytotoxicity of simvastatin-Cy5.5 in *KRAS*^{MUT} cell lines compared to simvastatin. Cell viability of CT26 and Panc1 cells after 24 h treatment with simvastatin-Cy5.5 (left, red) or unconjugated simvastatin (right, green) at concentrations of 0.1, 1, and 10 μM. While simvastatin-Cy5.5 induced dose-dependent cytotoxicity in *KRAS*^{MUT} cells, simvastatin alone showed minimal toxicity across the same dose range. Bars indicate mean ± S.E. ($n \geq 3$). Statistical comparisons were made against the control group using Student's t-test; *$p < 0.05$, **$p < 0.01$, ***$p < 0.001$.
