# Supplementary material for: Statin-dye conjugates for selective targeting of KRAS mutant cancer cells
Source: PLoS One. 2026 Jan 9;21(1):e0340189. doi: 10.1371/journal.pone.0340189 (PMC12788682; doi:10.1371/journal.pone.0340189)
Supplement: S11 Fig — Cell viability of Panc1 and CAF19 cells after 24 h treatment with pravastatin-Cy5.5 (left, red) and unconjugated pravastatin (right, gray) at concentrations of 0, 2, and 5 µM. Pravastatin-Cy5.5 exhibited dose-dependent cytotoxicity in Panc1, while showing little to no toxicity in CAF19 cells. In contrast, pravastatin alone had minimal effects across all conditions Bars indicate mean ± S.E. (n ≥ 3). Statistically significant differences are indicated by ** for p < 0.01 and *** for p < 0.001 (student t-test). (PDF) [file pone.0340189.s011.pdf]

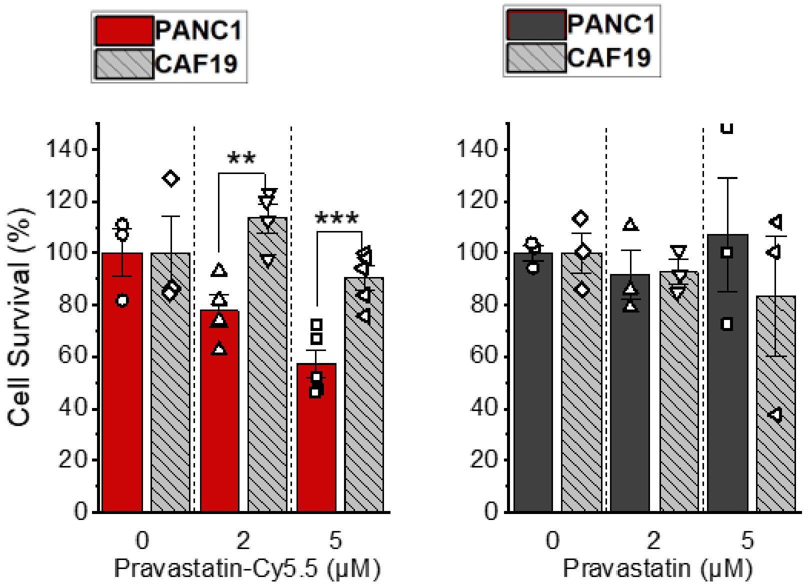

Figure S11. Selective cytotoxicity of pravastatin-Cy5.5 in *KRAS*^{MUT} cell line compared to pravastatin. Cell viability of Panc1 and CAF19 cells after 24 h treatment with pravastatin-Cy5.5 (left, red) and unconjugated pravastatin (right, gray) at concentrations of 0, 2, and 5 µM. Pravastatin-Cy5.5 exhibited dose-dependent cytotoxicity in Panc1, while showing little to no toxicity in CAF19 cells. In contrast, pravastatin alone had minimal effects across all conditions Bars indicate mean ± S.E. ($n \geq 3$). Statistically significant differences are indicated by ** for $p < 0.01$ and *** for $p < 0.001$ (Student's t-test).
