# Supplementary material for: Statin-dye conjugates for selective targeting of KRAS mutant cancer cells
Source: PLoS One. 2026 Jan 9;21(1):e0340189. doi: 10.1371/journal.pone.0340189 (PMC12788682; doi:10.1371/journal.pone.0340189)
Supplement: S12 Fig — Confocal fluorescence images showing intracellular localization of simvastatin-Rhodamine B (yellow) after 1 h incubation at 50 nM in DLD1 KRASWT and KRASMUT cells (top), HCT116 KRASWT and KRASMUT cells (middle), and MCF10A PTENWT and PTENKO cells (bottom). Nuclei were counterstained with DAPI (blue). Minimal intracellular fluorescence was observed across all cell types, indicating poor uptake of simvastatin-Rhodamine B and no apparent KRAS- or PTEN-dependent selectivity. (PDF) [file pone.0340189.s012.pdf]

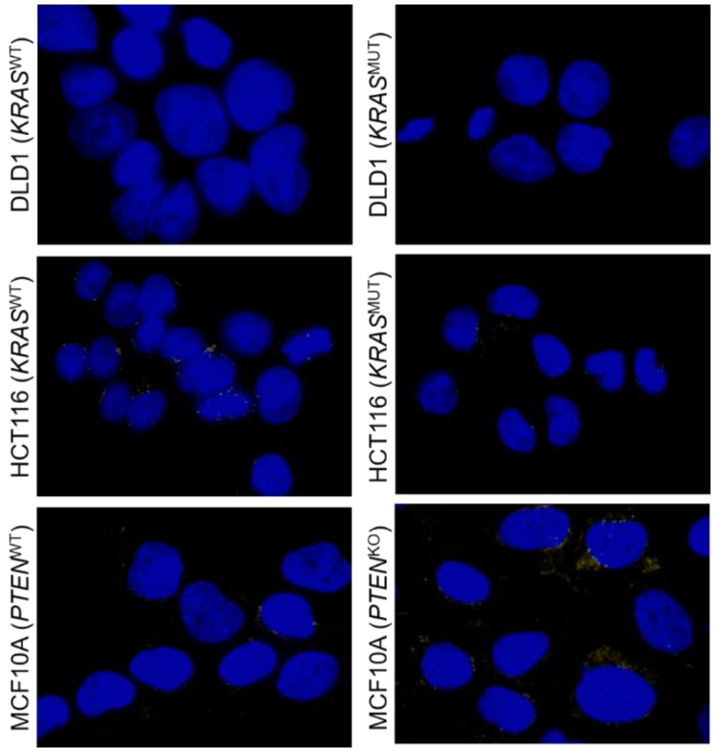

Figure S12. Cellular uptake of simvastatin-Rhodamine B in various cell lines. Confocal fluorescence images showing intracellular localization of simvastatin-Rhodamine B (yellow) after 1 h incubation at 50 nM in DLD1 *KRAS*^{WT} and *KRAS*^{MUT} cells (top), HCT116 *KRAS*^{WT} and *KRAS*^{MUT} cells (middle), and MCF10A *PTEN*^{WT} and *PTEN*^{KO} cells (bottom). Nuclei were counterstained with DAPI (blue). Minimal intracellular fluorescence was observed across all cell types, indicating poor uptake of simvastatin-Rhodamine B and no apparent *KRAS*- or *PTEN*-dependent selectivity.
