# Supplementary material for: Statin-dye conjugates for selective targeting of KRAS mutant cancer cells
Source: PLoS One. 2026 Jan 9;21(1):e0340189. doi: 10.1371/journal.pone.0340189 (PMC12788682; doi:10.1371/journal.pone.0340189)
Supplement: S13 Fig — The cell nuclei were stained with DAPI (blue). The scale bars indicate 100 μm. (PDF) [file pone.0340189.s013.pdf]

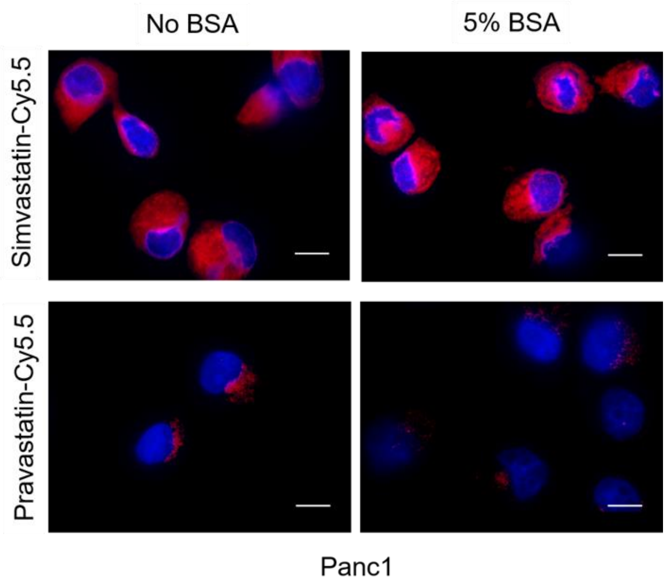

Figure S13. Uptake of simvastatin-Cy5.5 (red) and pravastatin-Cy5.5 (red) in Panc1 under no bovine serum albumin (BSA) and 5% BSA condition. The cell nuclei were stained with DAPI (blue). The scale bars indicate 100 µm.
