# Supplementary material for: Statin-dye conjugates for selective targeting of KRAS mutant cancer cells
Source: PLoS One. 2026 Jan 9;21(1):e0340189. doi: 10.1371/journal.pone.0340189 (PMC12788682; doi:10.1371/journal.pone.0340189)
Supplement: S1 Table — (PDF) [file pone.0340189.s014.pdf]

Cell line	Description	Mutation Status	Origin	Notes
Panc1	Pancreatic ductal adenocarcinoma	*KRAS*^{G12D}	Human	
BxPC3	Pancreatic ductal adenocarcinoma	*KRAS*^{WT}	Human	
HPDE *iKRAS*	Pancreatic duct epithelial cells	*KRAS*^{G12D} (inducible)	Human	Model for studying *KRAS* activation
MCF10A *PTEN*^{KO}	Breast epithelial cells	*KRAS*^{WT} *PTEN*^{KO}	Human	
MCF10A *PTEN*^{WT}	Breast epithelial cells	*KRAS*^{WT} *PTEN*^{WT}	Human	
CAF19	Cancer-associated fibroblasts	*KRAS*^{WT}	Human	
HCT116 *KRAS*^{MUT}	Colorectal adenocarcinoma	*KRAS*$^{WT/G13D}$	Human	
HCT116 *KRAS*^{WT}	Colorectal adenocarcinoma	*KRAS*^{WT}	Human	
DLD1 *KRAS*^{MUT}	Colorectal adenocarcinoma	*KRAS*$^{WT/G13D}$	Human	
DLD1 *KRAS*^{WT}	Colorectal adenocarcinoma	*KRAS*^{WT}	Human	

Table 1. Characteristics of the cells used in this study
